# Anaerobic bacteria: a neglected reservoir of mobile oxazolidinone resistance genes

**DOI:** 10.1080/19490976.2026.2734670

**Published:** 2026-09-18

**Authors:** Simeng Liu, Shuangyu Xie, Yongshan Song, Ruichao Li, Andrea Brenciani, Stefan Schwarz, Wanjiang Zhang, Yao Zhu

**Affiliations:** a State Key Laboratory of Animal Disease Control and Prevention, Harbin Veterinary Research Institute, Chinese Academy of Agricultural Sciences, Harbin, China; b Jiangsu Co-innovation Center for Prevention and Control of Important Animal Infectious Diseases and Zoonoses, College of Veterinary Medicine, Yangzhou University, Yangzhou, China; c Unit of Microbiology, Department of Biomedical Sciences and Public Health, Polytechnic University of Marche Medical School, Ancona, Italy; d Institute of Microbiology and Epizootics, Centre for Infection Medicine, School of Veterinary Medicine, Freie Universität Berlin, Berlin, Germany; e Veterinary Centre for Resistance Research (TZR), School of Veterinary Medicine, Freie Universität Berlin, Berlin, Germany

**Keywords:** Gut anaerobes, oxazolidinones, antimicrobial resistance, oxazolidinone resistance genes, mobile genetic elements

## Abstract

Oxazolidinones are critical last-resort antimicrobials against severe infections caused by Gram-positive bacteria, whereas mobile oxazolidinone resistance genes (MORGs, including *cfr*, *optrA*, *poxtA*) severely compromise their clinical efficacy. To date, most surveillance and mechanistic studies focusing on MORGs have focused on aerobic pathogens, while anaerobic commensal and opportunistic bacteria are largely overlooked as hidden resistance reservoirs, leading to critical knowledge gaps under the One Health framework. Here, we identified six MORG-carrying anaerobic gut bacteria belonging to four distinct genera. Notably, this is the first report on the identification of *cfr* (B) in Gram-negative bacteria and *optrA* in three previously unreported host species. Genomic dissection further confirmed diverse mobile genetic elements driving horizontal dissemination of MORGs across phylogenetically distant anaerobes. Multiple additional resistance genes coexisted with MORGs in these gut anaerobes. Our findings demonstrate that intestinal anaerobic bacteria constitute a neglected critical reservoir for clinically vital MORGs, highlighting an urgent need for strengthened antimicrobial resistance surveillance of anaerobes across animal and human hosts.

Anaerobic bacteria are an important component of the human and animal microbiota, and some of them are also significant opportunistic pathogens that can cause a variety of serious infections. In recent years, the increasing antimicrobial resistance (AMR) of anaerobes has become a major public health challenge.^1^ However, compared with aerobic bacteria, research on AMR of anaerobes remains very limited to date. In addition, due to the particularity of their culture conditions, their threat to public health has long been underestimated and neglected. More importantly, anaerobes are also considered a potential reservoir of AMR genes, which are capable of horizontal transfer across distinct bacterial pathogens.^2^

Oxazolidinones, including linezolid and tedizolid, are last-resort antimicrobial agents for combating life-threatening Gram-positive pathogens, including methicillin-resistant *Staphylococcus aureus* (MRSA), vancomycin-resistant enterococci (VRE), and penicillin-resistant *Streptococcus pneumoniae* (PRSP).^3^ In addition, linezolid also demonstrates moderate activity against various Gram-positive and Gram-negative anaerobes.^4^ Unfortunately, the emergence and rapid spread of mobile oxazolidinone resistance genes (MORGs), including *cfr*, *cfr*-like, *optrA,* and *poxtA*, represent a significant threat to public health and a growing global concern. To date, apart from *Clostridioides* and *Clostridium*, the prevalence of MORGs among anaerobic bacteria remains largely unexplored. To address this knowledge gap, we conducted epidemiological surveillance coupled with whole-genome sequencing (WGS) to characterize anaerobic bacteria harboring MORGs.

In 2025, a total of 83 fresh fecal samples were collected from three livestock species (chicken, pig, and dairy cattle) across 17 commercial farms located in five cities of Heilongjiang Province, China. Specifically, 38 samples were obtained from chickens, 26 from pigs, and 19 from dairy cattle (Table S1). These samples were inoculated into Anaerobic Meat Liver Broth to enrich anaerobic bacteria, followed by anaerobic incubation for 10 h. Subsequently, the bacterial suspension was streaked onto Gifu Anaerobic Medium agar containing linezolid (4 mg/L) and incubated anaerobically at 37 °C for 48 h. Single colonies with divergent morphological features were picked for subsequent purification and subculturing. The presence of MORGs was determined by PCR assays as previously described.^5^ Species identification was performed using 16S rRNA sequencing analysis. Data analysis was performed using SPSS v19.0.

A total of six anaerobic strains harboring MORGs were identified, including single strains of *Massilimicrobiota timonensis*, *Megasphaera elsdenii*, and *Paraclostridium bifermentans* as well as three *Fusobacterium mortiferum* strains (Table S2), yielding an isolation rate of 7.2%. All of them tested positive for the *optrA* gene, with the exception of the *M. timonensis* strain harboring the *cfr*(B) gene. Antimicrobial susceptibility testing (AST) was performed using agar dilution method according to CLSI recommendations.^6^ These six anaerobic strains exhibited a multidrug-resistance (MDR) phenotype, with high MICs of linezolid, chloramphenicol, florfenicol, erythromycin, and tetracycline. All six strains were susceptible to meropenem (Table S3). Subsequently, the six identified strains were subjected to WGS with Illumina NovaSeq and Oxford Nanopore PromethION platforms. *De novo* hybrid genome assembly was conducted with Unicycler v0.4.8 (https://github.com/rrwick/Unicycler). Annotation of the genome was performed using the online RAST tool (http://rast.nmpdr.org/), with subsequent manual correction. Antimicrobial resistance genes (ARGs) and plasmid replicon types were identified using ResFinder and PlasmidFinder tools available on the Center for Genomic Epidemiology (CGE) server (http://www.genomicepidemiology.org/). Linear comparisons of the genetic environments flanking resistance genes were visualized with Easyfig (https://mjsull.github.io/Easyfig/).

*M. timonensis* was firstly identified in the human gut microbiota in France in 2019.^7^ To date, this novel species has rarely been reported in subsequent studies. Of particular interest, the *cfr*(B) gene has previously been exclusively identified among Gram-positive bacteria, including *Clostridioides*, *Enterococcus*, and *Staphylococcus*.^8^ To the best of our knowledge, this is the first report of the *cfr*(B) gene in a naturally occurring Gram-negative bacterium. To investigate whether *cfr*(B) was actively transcribed in *M. timonensis* JX4, both reverse-transcription PCR (RT-PCR) and quantitative RT-PCR (qRT-PCR) assays were performed, and the results verified that *cfr*(B) was transcriptionally active (Fig. S1). To interpret the successful heterologous transcription of *cfr*(B) from a Gram-positive genetic background in the Gram-negative anaerobic bacterium *M. timonensis*, we systematically analyzed the upstream intergenic region of *cfr* (B). A canonical σ^70^ (RpoD) housekeeping promoter architecture was identified, featuring a -35 hexamer (TTTACA), a -10 Pribnow box (TATACT), and a 17-bp AT-rich spacer. Additionally, the putative Shine-Dalgarno (SD) sequence (AGGA), the core element of ribosome-binding site, preceding the *cfr*(B) start codon exhibited high complementarity to anti-SD (CCUCCU) at the 3′ end of the 16S rRNA of *M. timonensis*. Genetic environment analysis showed that the *cfr*(B) gene was located on a transposon-like genetic element resembling Tn*6218* in the chromosomal DNA of *M. timonensis* JX4. A comparative analysis found that this Tn*6218*-like structure exhibited high homology with the Tn*6218* transposon found in both *Clostridioides difficile* Ox2167 (HG002396.1) and *Enterococcus faecium* 448-18961R (KR610408.1) (Fig. S2). Collectively, these findings suggest that Tn*6218*/Tn*6218-*like transposons serve as vectors mediating the cross-species dissemination of the *cfr*(B) gene.

*M. elsdenii* has been detected in the intestinal contents and feces of humans, ruminants, and swine.^9^ Genome analysis revealed that the *optrA* gene was embedded in an IS*Sag10*-flanked pseudocompound transposon (PCT)-like structure within the chromosomal DNA of *M. elsdenii* strain YS46 (Fig. S3). It was found to be inserted into the *hsdm* gene encoding a site-specific DNA methyltransferase and an 8-bp target site duplication (5′-TTTGATAA-3′) was observed. As an indispensable component of the Type I restriction-modification (R-M) system, *hsdm* is responsible for host genome self-methylation.^10^ Insertional disruption of *hsdm* eliminates the HsdM methyltransferase function. Without proper methylation, the intact restriction subunit fails to target unmethylated exogenous DNA, disabling R-M-based immunity against horizontally transferred genetic material. Loss of R-M defense may increase cellular permissiveness to incoming mobile genetic elements (MGEs) and ARGs, favoring the accumulation, recombination, and spread of resistance genes. Furthermore, disruption of genomic-methylation patterns may compromise DNA replication, DNA repair, and chromosome segregation, leading to elevated rates of genomic mutation and homologous recombination.^11^ Of note, six copies of the *lnu*(C) lincosamide resistance gene were detected on the chromosome, all located within MTn*Sag1*-like transposon structures. These observations suggest that *M. elsdenii* may serve as a potential reservoir for lincosamide resistance. Notably, AST results revealed that no increase in lincosamide MICs was observed for *M. elsdenii* strain YS46 harboring five copies of *lnu*(C), relative to strain SYC45 carrying a single copy of *lnu*(C) (Table S3). Comparable observations have been documented for some other resistance genes, such as the colistin resistance gene *mcr-1* and the tigecycline resistance gene cluster *tmexC2D2.2-toprJ2*.^12^ ^,^ ^13^ Therefore, resistance phenotypes may be associated with multiple factors beyond gene dosage, including gene expression, host genetic background, regulatory context, and other resistance-associated mechanisms. Nevertheless, multiple *lnu* (C) copies facilitate stable maintenance of this resistance gene in the host isolate.

*Fusobacterium* is a genus that is widely distributed in the oral cavity and gastrointestinal tract of humans and animals.^14^ To date, only one published review has documented the presence of the *optrA* gene in the genome of the *F. hominis* strain NSJ-57 (CP060637.1), recovered from a human fecal sample in China.^4^ In the present study, we identified three *optrA*-positive *F. mortiferum* strains, representing the first report of the *optrA* gene in this bacterial species. Genome analysis showed that the *optrA* gene was chromosomally encoded in *F. mortiferum* strains SYC45 and SM100, while in strain FC91, it was carried by a novel MDR plasmid, designated pFC91-1 (Fig. S4). BLAST analysis using the complete sequence of plasmid pFC91-1 as a query revealed no significant homologous sequences. PlasmidFinder analysis indicated that plasmid pFC91-1 was non-typeable, while conjugative modules were identified in pFC91-1, implying that it was a conjugative plasmid. Filter mating experiments were performed using rifampicin-resistant *Enterococcus faecalis* JH2-2 as the recipient strain and revealed that plasmid pFC91-1 could transfer into *E. faecalis* JH2-2 at low conjugation efficiency (1.8 ± 0.5 × 10^−8^), confirming its conjugative capability. In the other two *F. mortiferum* strains, the chromosomally located *optrA* gene was found within a similar MDR region, which was inserted into distinct chromosomal loci (Fig. S5, S6). These observations revealed that the *optrA* gene can be horizontally transferred among *Fusobacterium* spp. via plasmids or other unidentified elements.

*P. bifermentans* is a Gram-positive bacterium that grows exclusively under anaerobic conditions.^15^ Genome analysis revealed that the *optrA* gene was located in a PCT-like structure bounded by two copies of IS*Vlu1* elements in the same orientation in the chromosomal DNA of *P. bifermentans* SY128. This structure shared high sequence identity with the corresponding segments of plasmids pQHY-2 (CP118266.1), unnamed1 (CP073071.1), and p21-D-5b (CP119189.1), all of which were from *C. perfringens* (Fig. S7). This IS*Vlu1*-flanked segment was found to be inserted into a AT-rich region and an 8-bp direct repeat (5′-AAAAAAAT-3′) was observed at the insertion site. A previous study indicated that such an IS*Vlu1*-flanked segment can form a translocatable unit (TU) that is capable of moving AMR genes.^16^ This observation revealed that the *optrA* gene in SY128 was likely obtained through TU-mediated horizontal gene transfer. In addition, conjugation assays showed that no transconjugants were obtained despite repeated attempts, likely due to the lack of functional conjugative transfer genes. Despite its inability to conjugatively transfer, this TU can be mobilized and disseminated between bacterial strains after being integrated into a conjugative plasmid or an integrative and conjugative element (ICE) carrying intact conjugation modules, highlighting its potential dissemination risk in anaerobic microbial communities.^17^

In conclusion, this study represents the first report of MORGs in *M. timonensis*, *M. elsdenii*, *P. bifermentans,* and *F. mortiferum*. Unexpectedly, the *optrA* gene is prevalent in various anaerobic bacteria, especially *Fusobacterium*, and co-existence of *optrA* with other resistance genes was commonly observed in multiple genera. In addition, our findings revealed that anaerobic bacteria represent an important potential reservoir for MORGs. These results emphasize the urgent need for enhanced AMR surveillance in anaerobic bacteria within a One Health framework.

## Supplementary Material

Supplementary Material Supplementary material.docx

## Data Availability

The genome sequences of all strains in this study were deposited in the NCBI database under BioProject accession number PRJNA1459018.
