# Supplementary material for: Anaerobic bacteria: a neglected reservoir of mobile oxazolidinone resistance genes
Source: Gut Microbes. 2026 Sep 18;18(1):2734670. doi: 10.1080/19490976.2026.2734670 (PMC13596870; doi:10.1080/19490976.2026.2734670)
Supplement: Supplementary file 1 [file KGMI_A_2734670_SM2254.docx]

**Table S1.** The details of the 83 fecal samples

| **Sample** | **Host** | **Geographic location** | **Farm** |
| --- | --- | --- | --- |
| Sample_1 | Chicken | Harbin City | Farm NPS |
| Sample_2 | Chicken | Harbin City | Farm NPS |
| Sample_3 | Chicken | Harbin City | Farm NPS |
| Sample_4 | Chicken | Harbin City | Farm NPS |
| Sample_5 | Chicken | Harbin City | Farm NPS |
| Sample_6 | Chicken | Harbin City | Farm NPS |
| Sample_7 | Chicken | Harbin City | Farm NPS |
| Sample_8 | Chicken | Harbin City | Farm FC |
| Sample_9 | Chicken | Harbin City | Farm FC |
| Sample_10 | Chicken | Harbin City | Farm FC |
| Sample_11 | Chicken | Harbin City | Farm FC |
| Sample_12 | Chicken | Harbin City | Farm FC |
| Sample_13 | Chicken | Daqing City | Farm TJ |
| Sample_14 | Chicken | Daqing City | Farm TJ |
| Sample_15 | Chicken | Daqing City | Farm TJ |
| Sample_16 | Chicken | Daqing City | Farm TJ |
| Sample_17 | Chicken | Daqing City | Farm TJ |
| Sample_18 | Chicken | Daqing City | Farm TJ |
| Sample_19 | Chicken | Daqing City | Farm SM |
| Sample_20 | Chicken | Daqing City | Farm SM |
| Sample_21 | Chicken | Daqing City | Farm SM |
| Sample_22 | Chicken | Daqing City | Farm SM |
| Sample_23 | Chicken | Mudanjiang City | Farm KUS |
| Sample_24 | Chicken | Mudanjiang City | Farm KUS |
| Sample_25 | Chicken | Mudanjiang City | Farm KUS |
| Sample_26 | Chicken | Mudanjiang City | Farm KUS |
| Sample_27 | Chicken | Mudanjiang City | Farm KUS |
| Sample_28 | Chicken | Suihua City | Farm SYC |
| Sample_29 | Chicken | Suihua City | Farm SYC |
| Sample_30 | Chicken | Suihua City | Farm SYC |
| Sample_31 | Chicken | Suihua City | Farm SYC |
| Sample_32 | Chicken | Suihua City | Farm SYC |
| Sample_33 | Chicken | Qiqihar City | Farm JX |
| Sample_34 | Chicken | Qiqihar City | Farm JX |
| Sample_35 | Chicken | Qiqihar City | Farm JX |
| Sample_36 | Chicken | Qiqihar City | Farm JX |
| Sample_37 | Chicken | Qiqihar City | Farm JX |
| Sample_38 | Chicken | Qiqihar City | Farm JX |
| Sample_39 | Pig | Harbin City | Farm SHF |
| Sample_40 | Pig | Harbin City | Farm SHF |
| Sample_41 | Pig | Harbin City | Farm SHF |
| Sample_42 | Pig | Harbin City | Farm BF |
| Sample_43 | Pig | Harbin City | Farm BF |
| Sample_44 | Pig | Harbin City | Farm BF |
| Sample_45 | Pig | Harbin City | Farm BF |
| Sample_46 | Pig | Harbin City | Farm BF |
| Sample_47 | Pig | Daqing City | Farm SRT |
| Sample_48 | Pig | Daqing City | Farm SRT |
| Sample_49 | Pig | Daqing City | Farm SRT |
| Sample_50 | Pig | Suihua City | Farm ZDX |
| Sample_51 | Pig | Suihua City | Farm ZDX |
| Sample_52 | Pig | Suihua City | Farm ZDX |
| Sample_53 | Pig | Suihua City | Farm ZDX |
| Sample_54 | Pig | Suihua City | Farm ZDX |
| Sample_55 | Pig | Suihua City | Farm ZDX |
| Sample_56 | Pig | Qiqihar City | Farm YS |
| Sample_57 | Pig | Qiqihar City | Farm YS |
| Sample_58 | Pig | Qiqihar City | Farm YS |
| Sample_59 | Pig | Qiqihar City | Farm YS |
| Sample_60 | Pig | Qiqihar City | Farm YS |
| Sample_61 | Pig | Qiqihar City | Farm YS |
| Sample_62 | Pig | Qiqihar City | Farm DBN |
| Sample_63 | Pig | Qiqihar City | Farm DBN |
| Sample_64 | Pig | Qiqihar City | Farm DBN |
| Sample_65 | Dairy cattle | Harbin City | Farm GGK |
| Sample_66 | Dairy cattle | Harbin City | Farm GGK |
| Sample_67 | Dairy cattle | Harbin City | Farm GGK |
| Sample_68 | Dairy cattle | Harbin City | Farm GGK |
| Sample_69 | Dairy cattle | Harbin City | Farm GGK |
| Sample_70 | Dairy cattle | Harbin City | Farm GGK |
| Sample_71 | Dairy cattle | Mudanjiang City | Farm HBA |
| Sample_72 | Dairy cattle | Mudanjiang City | Farm HBA |
| Sample_73 | Dairy cattle | Mudanjiang City | Farm HBA |
| Sample_74 | Dairy cattle | Mudanjiang City | Farm HBA |
| Sample_75 | Dairy cattle | Daqing City | Farm SY |
| Sample_76 | Dairy cattle | Daqing City | Farm SY |
| Sample_77 | Dairy cattle | Daqing City | Farm SY |
| Sample_78 | Dairy cattle | Daqing City | Farm SY |
| Sample_79 | Dairy cattle | Daqing City | Farm SY |
| Sample_80 | Dairy cattle | Daqing City | Farm SY |
| Sample_81 | Dairy cattle | Qiqihar City | Farm PWM |
| Sample_82 | Dairy cattle | Qiqihar City | Farm PWM |
| Sample_83 | Dairy cattle | Qiqihar City | Farm PWM |

**Table S2.** Characteristics of six anaerobic strains harbouring oxazolidinone resistance genes

| **Strain** | **Species** | **Source** | **Gram Staining** | **AMR genes** | **Location of oxazolidinone resistance gene** | **GenBank Accession number** |
| --- | --- | --- | --- | --- | --- | --- |
| JX4 | *Massilimicrobiota timonensis* | Chicken | Negative | ***cfr*(B)**, *tet*(44) | Chromosome | SAMN57539665 |
| YS46 | *Megasphaera elsdenii* | Pig | Negative | *bla*_ACI-1_, *lnu*(C), ***optrA***, *fexA*, *tet*(O/W)-2, *tet*(L) | Chromosome | SAMN57540146 |
| SYC45 | *Fusobacterium mortiferum* | Chicken | Negative | *erm*(A), *lnu*(C), ***optrA***, *fexA*, *cat*(pC194), *tet*(O) | Chromosome | SAMN57540259 |
| SM100 | *Fusobacterium* *mortiferum* | Chicken | Negative | *aph(2'')-If*, *bla*_OXA-85_, *erm*(A), ***optrA***, *fexA*, *cat*(pC194), *tet*(O), *tet*(L), *tetA*(P) | Chromosome | SAMN57540307 |
| FC91 | *Fusobacterium mortiferum* | Chicken | Negative | *bla*_OXA-85_, *lnu*(C), *erm*(A), ***optrA***, *cat*(pC194), *fexA*, *tet*(O), *tetA*(P), *tet*(L) | Plasmid | SAMN57540719 |
| SY128 | *Paraclostridium bifermentans* | Dairy cattle | Positive | *erm*(A), ***optrA***, *fexA*, *tetA*(P) | Chromosome | SAMN57540724 |

**Table S3.** MIC_S_ of six anaerobic strains harboring MORGs

| Antimicrobial agents | MIC breakpoints (mg/L) according to CLSI* | | | MIC (mg/L) | | | | | |
| --- | --- | --- | --- | --- | --- | --- | --- | --- | --- |
|  | S | I | R | JX4 | YS46 | SYC45 | SM100 | FC91 | SY128 |
| Linezolid | - | - | - | >16 | >16 | >16 | >16 | >16 | 16 |
| Chloramphenicol | ≤8 | 16 | ≥32 | 32 | >64 | >64 | 64 | >64 | 64 |
| Florfenicol | - | - | - | 32 | >64 | >64 | 32 | >64 | 32 |
| Erythromycin | - | - | - | >64 | >64 | >64 | >64 | >64 | >64 |
| Clindamycin | ≤2 | 4 | ≥8 | 64 | 32 | 64 | 4 | 64 | 8 |
| Lincomycin | - | - | - | >256 | >256 | >256 | 16 | >256 | 32 |
| Meropenem | ≤4 | 8 | ≥16 | 2 | ≤0.5 | ≤0.5 | 4 | 4 | ≤0.5 |
| Tetracycline | ≤4 | 8 | ≥16 | >64 | 32 | 16 | 32 | 32 | >64 |

* CLSI document M100, 35th edition

- no breakpoints available.

**Figure S1.** (a) RT-PCR detection of *cfr*(B) transcript in *M. timonensis* JX4. Lane 1: Positive control using genomic DNA as template; Lane 2: Negative control (sterile water); Lane 3: DNase-treated RNA sample (no-reverse-transcription control); Lane 4: cDNA template reversely transcribed from total RNA. Marker: DNA molecular-weight marker. (b) qRT-PCR detection of *cfr*(B) transcript in *M. timonensis* JX4. Blue, genomic DNA samples; red, cDNA samples; orange, DNase-treated RNA samples; green, negative controls. Each group was performed in triplicate.

**Figure S2.** Schematic presentation and comparison of the genetic environment of *cfr*(B) in *M. timonensis* JX4 with that in *C. difficile* strain Ox2167 and *E. faecium* strain 448-18961R. The positions and transcriptional directions of the ORFs are denoted with arrows. The *cfr*(B) gene is marked in red.

**Figure S3.** Schematic presentation and comparison of the genetic environment of *optrA* in *M. elsdenii* strain YS46 with that in *F. hominis* strain NSJ-57. The positions and transcriptional directions of the ORFs are denoted with arrows. ORFs of different functions are presented in various colors. Genes are color-coded as follows: green, mobile element; red, antimicrobial resistance; orange, other proteins. The truncated *hsdm* gene is marked in yellow. DR represents direct repeat sequences.

**Figure S4.** Circular representation of the plasmid pFC91-1. The circles show (from outside to inside): predicted coding sequences, GC skew, GC content and scale in kb. ORFs of different functions are presented in various colours. Genes are color-coded as follows: purple, replication; green, mobile element; red, antimicrobial resistance; blue, conjugational transfer; orange, other proteins.

**Figure S5.** Schematic presentation and comparison of the genetic environment of *optrA* in *F. mortiferum* strain SYC45 with that in *F. hominis* strain NSJ-57. The positions and transcriptional directions of the ORFs are denoted with arrows. ORFs of different functions are presented in various colors. Genes are color-coded as follows: green, mobile element; red, antimicrobial resistance; orange, other proteins. DR represents direct repeat sequences.

**Figure S6.** Schematic presentation and comparison of the genetic environment of *optrA* in *F. mortiferum* strain SM100 with that in *F. hominis* strain NSJ-57. The positions and transcriptional directions of the ORFs are denoted with arrows. ORFs of different functions are presented in various colors. Genes are color-coded as follows: green, mobile element; red, antimicrobial resistance; orange, other proteins.

**Figure S7.** Schematic presentation and comparison of the genetic environment of *optrA* in *P. bifermentans* strain SY128 with that in plasmid pQHY-2. The positions and transcriptional directions of the ORFs are denoted with arrows. ORFs of different functions are presented in various colors. Genes are color-coded as follows: green, mobile element; red, antimicrobial resistance; orange, other proteins. DR represents direct repeat sequences.
